# Modifying MSC Phenotype to Facilitate Bone Healing: Biological Approaches

**DOI:** 10.3389/fbioe.2020.00641

**Published:** 2020-06-24

**Authors:** Stuart B. Goodman, Tzuhua Lin

**Affiliations:** ^1^Department of Orthopaedic Surgery, Stanford University School of Medicine, Redwood City, CA, United States; ^2^Department of Bioengineering, Stanford University, Stanford, CA, United States; ^3^Orthopaedic Research Laboratories, Stanford University, Stanford, CA, United States

**Keywords:** mesenchymal stem cell, mesenchymal stromal cell, inflammation, preconditioning, hypoxia, genetic manipulation

## Abstract

Healing of fractures and bone defects normally follows an orderly series of events including formation of a hematoma and an initial stage of inflammation, development of soft callus, formation of hard callus, and finally the stage of bone remodeling. In cases of severe musculoskeletal injury due to trauma, infection, irradiation and other adverse stimuli, deficient healing may lead to delayed or non-union; this results in a residual bone defect with instability, pain and loss of function. Modern methods of mechanical stabilization and autologous bone grafting are often successful in achieving fracture union and healing of bone defects; however, in some cases, this treatment is unsuccessful because of inadequate biological factors. Specifically, the systemic and local microenvironment may not be conducive to bone healing because of a loss of the progenitor cell population for bone and vascular lineage cells. Autologous bone grafting can provide the necessary scaffold, progenitor and differentiated lineage cells, and biological cues for bone reconstruction, however, autologous bone graft may be limited in quantity or quality. These unfavorable circumstances are magnified in systemic conditions with chronic inflammation, including obesity, diabetes, chronic renal disease, aging and others. Recently, strategies have been devised to both mitigate the necessity for, and complications from, open procedures for harvesting of autologous bone by using minimally invasive aspiration techniques and concentration of iliac crest bone cells, followed by local injection into the defect site. More elaborate strategies (not yet approved by the U.S. Food and Drug Administration-FDA) include isolation and expansion of subpopulations of the harvested cells, preconditioning of these cells or inserting specific genes to modulate or facilitate bone healing. We review the literature pertinent to the subject of modifying autologous harvested cells including MSCs to facilitate bone healing. Although many of these techniques and technologies are still in the preclinical stage and not yet approved for use in humans by the FDA, novel approaches to accelerate bone healing by modifying cells has great potential to mitigate the physical, economic and social burden of non-healing fractures and bone defects.

## Introduction

Complex fractures and bone defects due to musculoskeletal trauma, infection, irradiation, tumor excision, periprosthetic osteolysis, and other etiologies do not always heal without intervention. In the USA alone, non-union constitutes ~1.9 to 10% of fractures and number ~100,000 cases per year (Thomas and Kehoe, 2020). Systemic factors that depress bone healing include specific medical conditions (e.g., chronic renal disease, diabetes, obesity, anemia, and others), older age, hormonal deficiency (e.g., hypothyroidism), poor nutrition, excessive alcohol use, smoking, and medications that interfere with bone formation or remodeling such as certain cancer chemotherapeutic agents and biologics for treating rheumatoid arthritis, non-steroidal anti-inflammatory drugs (NSAIDs), corticosteroids, some anti-coagulants, and others (Giannoudis et al., 2007; Santolini et al., 2015; Zura et al., 2016; Thomas and Kehoe, 2020). Autologous bone graft (ABG) is the gold standard to obtain healing of bone defects and fracture non-union (Sen and Miclau, 2007). ABG contains all elements for bone regeneration including a calcified collagen-based scaffold, viable differentiated, and progenitor cells of the mesenchymal and vascular lineages, and the appropriate proteins and other factors that function as biological cues for cell-guided matrix deposition. Non-union in the elderly patient is particularly challenging, and will only increase due to the aging of our general population; in the USA, individuals over 65 years of age are estimated to double in about 30 years, from 48 million in 2015 to 88 million by 2050 (Wan et al., 2016). The elderly comprises about 13% of the population in the USA, however admissions to hospitals by the elderly, mostly for fracture care, constitute 50% of all cases (Wagner et al., 2019). Elderly patients present a unique problem, because the bone graft harvested from the iliac crest and other accessible areas is often deficient in the quality and quantity of bone (Hobby and Lee, 2013). Furthermore, mesenchymal stem cells (MSCs) from elderly patients demonstrate reduced proliferative capacity, chemotactic ability, and the potential for differentiation (Wagner et al., 2019). Similarly, endothelial progenitor cells also show decreased proliferation, migration and function.

Given the above challenges to obtain bone union in patient populations with different demographics and co-morbidities, novel strategies must be devised to augment or replace autologous bone. Potential approaches include the use of improved scaffolds and addition of exogenous growth factors; however, the most difficult aspect of this equation is to renew or revitalize the host cells locally or provide additional cells of the MSC-osteoblast cell lineage, the endothelial cell lineage, and other cells that could engraft or provide critical signaling mechanisms to enhance bone healing (Prockop, 2009). Although there has been extensive literature on the use of bone substitutes, novel scaffolds and growth factors, studies focused on providing or augmenting the deficient cellular components that are needed for bone healing have received less attention (Bravo et al., 2013; Park et al., 2018; Marongiu et al., 2020; Pereira et al., 2020).

The current review summarizes the latest *in vitro* and *in vivo* research on the manipulation of the cellular elements, focusing on MSCs, to be grafted directly into an area of bone deficiency or fracture non-union to enhance bone formation and in some instances, decrease bone degradation. Although the majority of these technologies are in the preclinical stage, the opportunities are far-reaching. To become a mainstay in the clinician's armamentarium in the future, these tools need to be thoroughly validated, and shown to be safe, efficacious and cost-effective (Gomez-Barrena et al., 2015).

One issue immediately comes to the forefront: should the medical practitioner replenish the deficient bone tissue using autologous or allogeneic cell grafting? As a general rule in any medical or surgical procedure, if there are cells or tissues available of sufficient number and quality in the host that are potentially usable with known and limited morbidity, this is normally the first option chosen. Autologous grafts are derived from the patient's own tissues; thus, these cells are non-immunogenic and will not transmit potential diseases that may be harbored by the donor (Dimitriou et al., 2011a; Egol et al., 2015; Nauth et al., 2015). However, harvesting of cells or tissues from the host takes time and therefore has an associated cost and potential morbidity (Dimitriou et al., 2011b; Hernigou P. et al., 2014; Egol et al., 2015). Furthermore, especially for larger bone defects, there may be autologous tissues or cells of insufficient quality or quantity for healing. Allogeneic tissues or in the present discussion, cells are harvested from another individual and processed under strict sterile and regulatory conditions. These cells may potentially transmit diseases, known or unknown to the host; the desired cell population(s) are usually selected and expanded, and packaged by the manufacturer prior to delivery. In addition to the potential transmission of disease and cost, when discussing MSCs, there are recent reports challenging their previously touted immune-privileged nature (Griffin et al., 2010, 2013; Ankrum et al., 2014; Berglund et al., 2017; Almeida-Porada et al., 2020). Autologous concentrated marrow cell aspirates or techniques such as the use of the reamer-aspirator also contain many different and important cell lineages and populations, as well as other factors that may enhance bone healing to a greater degree than a graft composed of a single cell lineage (Henrich et al., 2010; Sagi et al., 2012; Seebach et al., 2015). This topic of discussion has yet to be resolved.

Although this review will focus on MSCS, all tissues require a robust vascular supply to maintain sufficient amounts of oxygen and nutrients, and rid the tissues of toxic waste. These concepts are also relevant to fracture healing and bone regeneration (Lee et al., 2008; Giles et al., 2017; Bahney et al., 2019). Endothelial progenitor cells are found in aspirates of the iliac crest, and in other sources commonly harvested for bone graft, however the numbers of endothelial colony-forming units (ECFCs) from these sources is very low (Pittenger et al., 1999). ECFCs, also called late outgrowth progenitor cells (late EPCs) come from progenitor cells in the peripheral or umbilical cord blood, and are phenotypic and functional precursors for cells of the endothelial lineage (Tasev et al., 2016). Early outgrowth progenitor cells (early EPCs) originate from the myeloid-monocytic lineage, are CD14^+^ and CD45^+^ and function mainly in a paracrine manner. ECFCs provide cells that incorporate into the endothelial lining of newly formed blood vessels. Combinations of ECFCs and MSCs or Adipose-derived stem cells (ASCs) are even more potent in neovascularization than ECFCs alone (Lin et al., 2012). Although ECFCs have been used extensively in scenarios of compromised vascularity and ischemia, there are also opportunities to use these cells in conjunction with MSCs for fracture healing and the regeneration of bone (Liu et al., 2012, 2013; Zigdon-Giladi et al., 2015; Sun et al., 2016; Tasev et al., 2016; Giles et al., 2017; Grosso et al., 2017).

## Methods of Cell Harvesting

### General Comments Concerning Cell Sources

Traditionally, bone graft for the purposes of obtaining union of fractures or healing of bone defects was harvested from the anterior or posterior iliac crest, because this area contained an abundance of all of the elements for osteogenesis. Other areas for obtaining bone graft are sometimes used, especially when working in local areas, such as the spine during decompression and fusion, greater trochanter, proximal or distal tibia, humerus etc. As an alternative, the reamer aspirator device can be used to harvest the contents of the medullary canal of long bones. The resultant aspirate has excellent regenerative capacity for bone healing, equivalent or in some studies superior to that of iliac crest graft (Henrich et al., 2010; Sagi et al., 2012; Seebach et al., 2015). Furthermore, aspirate from the reamer device can be used for grafting of large critical sized defects (Egol et al., 2015).

An alternative source of cells for bone healing is fat, which is usually abundant and can be accessed via liposuction. Fat is composed of ~90% mature fat cells and ~10% of a stromal vascular fraction (SVF). The SVF is composed of a heterogeneous population of cells including fibroblasts, vascular smooth muscle cells and pericytes, endothelial cells, monocyte/macrophages, lymphocytes, ASCs, and other precursor cells. It has been reported that up to 1 X 10^7^ ASCs can be isolated from ~300 cc of fat aspirate (Romagnoli and Brandi, 2014). ASCs have been reported to have very similar properties to bone marrow derived MSCs, although there are differences in some of the cell surface markers such as adhesion molecules (De Ugarte et al., 2003). ASCs are capable of differentiation into mesenchymal-based cells such as adipocytes, osteoblasts, chondrocytes, myocytes, and other cells (Tajima et al., 2018). The fat-derived cell population can be used either as a point-of care product or processed further to isolate, expand and concentrate the desired cell population (Romagnoli and Brandi, 2014; Grayson et al., 2015; Alt et al., 2020). Studies have shown that differentiated ASC-derived osteoblast lineage cells are effective in forming bone and healing bone defects (Parrilla et al., 2011; Mizuno et al., 2012; Tajima et al., 2018). Several recent reviews have summarized the preclinical and clinical data relevant to the use of ASCs for bone healing (Romagnoli and Brandi, 2014; Grayson et al., 2015; Tajima et al., 2018). Despite the known efficacy of ASCs for healing of bone, the use of this source is uncommon in orthopaedic surgery and is more common in the plastic surgery, perhaps because of the concurrent liposuction procedure.

### Harvesting From the Iliac Crest

Autologous bone graft harvested from the iliac crest is the gold standard by which other sources and techniques are measured. The technique of cell harvesting for the purposes of grafting of bone defects, non-union, osteonecrosis and other bone deficiencies has been well-described (Hernigou et al., 2005, 2015; Piuzzi et al., 2018). The technique should be mastered with a clear understanding of how to accomplish the technique safely, according to considerations of pelvic anatomy (Hernigou J. et al., 2014). Although some will report that red blood cells (RBCs) constitute the majority of cells in the marrow, by definition, RBCs are not cells because they do not possess a nucleus. The same objection could be made for platelets. It should be recognized that the majority of “true” cells in the iliac crest are not progenitor cells for osteoblasts or endothelial cells. The pelvic marrow is mostly composed of myelopoietic white blood cells (about 50%), erythropoietic cells (25%) and lymphocytic lineage cells, in a stroma containing fibroblasts, adipocytes, osteoblasts, osteoclasts, and endothelial cells. With aging, the normally “red” marrow becomes “yellow,” due to a predominance of adipocytes. Colony forming unit-fibroblast (CFU-F) cells that are precursors of the mesenchymal stem cell lineage are rare, and constitute approximately one out of every 30,000+ nucleated cells harvested from the anterior iliac crest, or ~600–1,200 progenitor cells per milliliter (cc) of aspirated unconcentrated bone marrow (Muschler et al., 1997; Hernigou et al., 2005, 2015). Repeated aspiration from the same location further dilutes the number of nucleated cells harvested, because the void quickly fills with red blood cells and plasma, that are less dense compared to the more densely packed cellular elements (Batinic et al., 1990; Muschler et al., 1997). It is therefore recommended that only 2 milliliters (ml) of marrow be aspirated in any one location, prior to repositioning the needle to another location. This can be done through the same insertion point or using another point of entry into the bone. Additionally, there are age-related and gender-related differences in the number of nucleated cells harvested. Muschler et al. noted that the number of nucleated cells in aspirates from elderly individuals (age 70 or older) are 50% (or substantially) less than those from teenagers (Muschler et al., 2001). Furthermore, the number of CFU-F cells derived from these nucleated cells was proportionally the same for aged men but was significantly less for elderly woman. The autologous iliac crest cell aspirates are gathered into heparinized syringes to avoid clotting.

Because of the paucity of cells, especially progenitor cells, in the aspirates, a method of concentration of the nucleated cells is normally used prior to local injection. Several studies have demonstrated that the desired outcome, namely fracture union or bone healing, is directly proportional to the number and concentration of progenitor cells that are injected locally (Hernigou and Beaujean, 2002; Hernigou et al., 2005, 2009). Concentration of the aspirated marrow also decreases the eventual volume that is injected locally, usually into a very confined space.

One classification system for describing different methods of cell separation and isolation of MSCs is as follows: (a) cell adherence to plastic surfaces; (b) gradient centrifugation methods; (c) membrane filtration methods; (d) Fluorescently labeled antibodies that bind to surface or intracellular molecules; and e. magnetically labeled antibodies that bind to surface molecules (Nicodemou and Danisovic, 2017). These methods all have their strengths and limitations. To maximize efficiency and minimize cost, the delivery of autologous byproducts, whether cells or biologics or both, would be optimized if these substances were delivered at the point-of-care, i.e., when the fracture, non-union or bone defect was undergoing additional invasive procedures such as surgical stabilization. Thus, centrifugation is the method that is most commonly used to concentrate the nucleated cell portion of bone aspirates for management of bone defects. Centrifugation separates different fluidic composites based on their differential densities. Centrifugation may be combined with the use of Ficoll, Ficoll-Paque, or other density gradient media or devices for cell separation. The above methods disperse and isolate the components of the marrow aspirate into various layers based on their density; the layer containing the mononuclear cells is called the buffy coat. The buffy coat contains a higher percentage of osteoprogenitor cells than in the harvested uncentrifuged marrow. Although the exact degree of concentration is controversial, most systems state that the level of concentration is ~2X-7X (Hegde et al., 2014; Dragoo and Guzman, 2020). There is also some controversy as to which commercial aspiration and concentration system is the optimal one for clinical use. The results of these systems are generally very similar (Hegde et al., 2014; Dragoo and Guzman, 2020). However, it must be emphasized that the manufacturers' instructions should be followed carefully to optimize cell retrieval. There are other techniques, often more expensive and/or limited in availability, to concentrate the buffy coat or subsets thereof, including fluorescence-activated cell sorting (FACS), selective retention that uses a special device incorporating a semipermeable membrane for cell selection based on their surface markers, magnetic separation using hyaluronan surface markers, the use of active and passive microfluidic devices, buoyancy activated cell sorting and others (Muschler et al., 2005; Caralla et al., 2012, 2013; Joshi et al., 2015).

## Methods of Cell Expansion and Selection

One issue that needs to be addressed initially pertains to the cell type(s) that the clinician would want to select for injection/grafting into a defect to enhance bone healing. Osteoblasts are differentiated cells that do not divide. Thus, a more logical option for regeneration of bone is to revert to an earlier stage in the lineage, such as the pre-osteoblast or the MSC. In preclinical studies, and in limited clinical trials (Gomez-Barrena et al., 2015, 2020; Lee et al., 2019; Hutchings et al., 2020), MSCs have been the target cell for isolation. As outlined above, MSCs are rare in the bone marrow; the approach most commonly used is expansion *in vitro*, and then injection or open grafting in a suitable carrier. This technique for bone regeneration is being performed outside of the USA, because it constitutes more than “minimal manipulation” of cells (U. S. Food and Drug Administration, 2017).

The definition of an MSC is controversial (Caplan, 2017a,b). Indeed, the International Society for Cell and Gene Therapy (ISCT) has distinguished the two terms mesenchymal stem cell and mesenchymal stromal cell (Viswanathan et al., 2019). In a position statement on nomenclature published in 2005 and updated in 2019, the ISCT states (*author: bracketed references omitted*): “The former (author: i.e., *mesenchymal stem cell*) refers to a stem cell population with demonstrable progenitor cell functionality of self-renewal and differentiation, whereas the latter (*author:* i.e., *mesenchymal stromal cell*) refers to a bulk population with notable secretory, immunomodulatory and homing properties.” (Horwitz et al., 2005; Viswanathan et al., 2019). Furthermore, they stated: “a minimal criteria to define multipotent MSCs as being plastic adherent, expressing CD73, CD90, and CD105, lacking the expression of hematopoietic and endothelial markers CD11b, CD14, CD19, CD34, CD45, CD79a, and HLA-DR and capable of *in vitro* differentiation into adipocyte, chondrocyte and osteoblast lineages” (Dominici et al., 2006). In the latest definition, the ISCT has endorsed continued use of the term MSC (Mesenchymal Stromal Cell) but recommends that (1) the tissue source or origin of the cells be clearly specified, (2) functional definitions must clarify whether one is referring to mesenchymal stromal cells or mesenchymal stem cells, (3) the term mesenchymal stromal cell be used to describe bulk unfractionated cell populations to recognize the fact that this may include other cell types, but not hematopoietic or endothelial cells (Viswanathan et al., 2019). This discussion is even more complex, due to the recent description of the Mouse Skeletal Stem Cell and the Human Skeletal Stem Cell (hSSC) (Chan et al., 2018; Gulati et al., 2018). The hSSC is defined as a self-renewing multipotent skeletal stem cell that is PDPN^+^CD146^−^CD73^+^CD164^+^, and generates progenitors of bone, cartilage, and stroma, but not fat.

**Figure 1:**
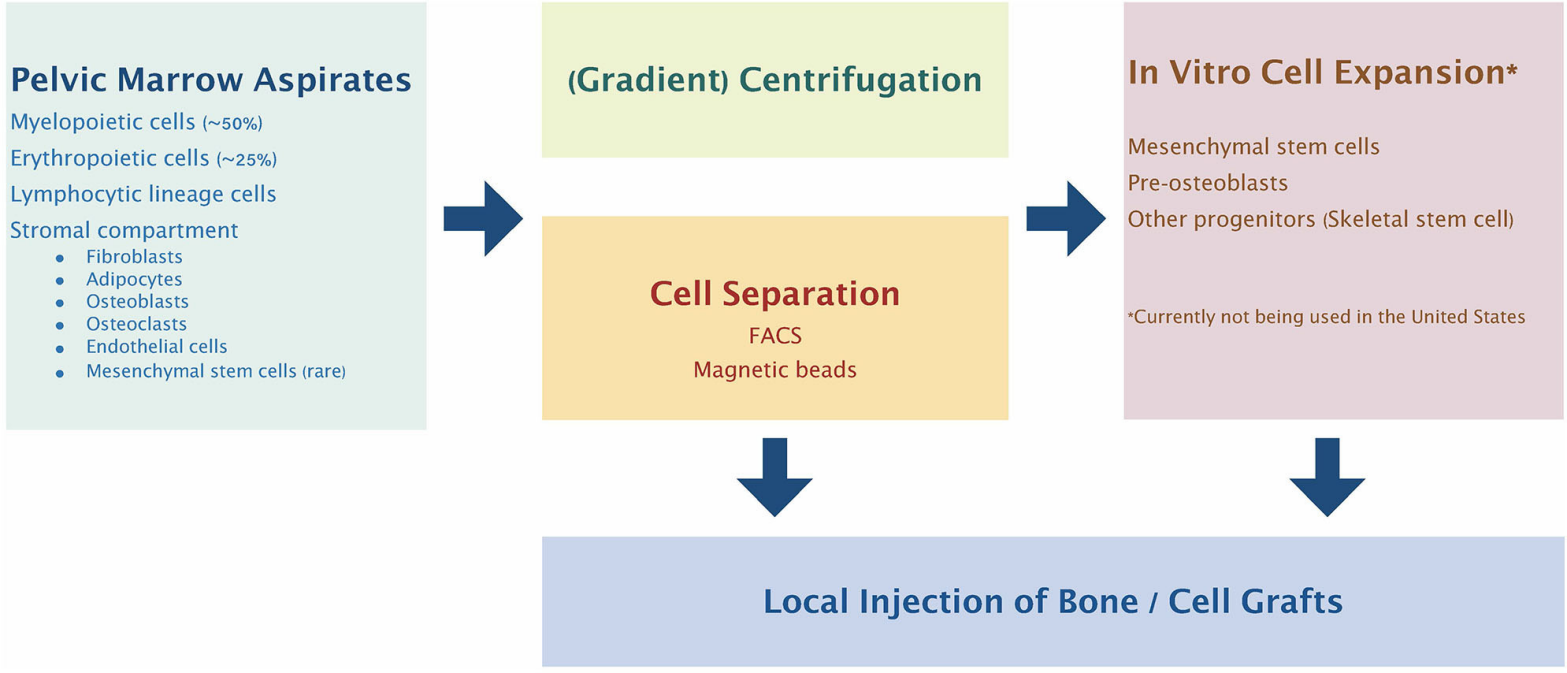
Bone marrow cell harvesting, selection, and expansion process most commonly used clinically. Pelvic marrow aspirates are concentrated by centrifugation and/or selection of specific cell populations before injection into the bone defect site. Alternatively, the selected progenitor cells can be expanded and manipulated *in vitro* to further enhance their therapeutic potential.

Given the above controversy, there have been many different methods for isolation of what the authors describe as mesenchymal stem cells (Kanczler et al., 2019) (Figure 1). One such methodology for GMP compliant generation of bone marrow derived MSCs and expansion on a large scale for a multicenter study of fracture healing in Europe has been described in detail (Fekete et al., 2012; Rojewski et al., 2013; Gomez-Barrena et al., 2020). This and other studies using MSCs are ongoing. However, it must be emphasized that such studies must be interpreted in light of the particular cell source, isolation and identification protocols, methods and details of cell delivery, the particular application for which these cells are given, and the methods of assessment of outcomes.

One further point needs emphasis. It is clear that MSCs interact with many other cells in the hematopoietic and mesenchymal lineages. In fact, there is substantial evidence that interactions with macrophages, T cells and other cells in the hematopoietic lineage are critical to the preconditioning and activation of MSCs and provide important cues and guides for their immunomodulatory function (see below). Thus, it might be prudent to consider not just the delivery of MSCs alone, but combinations of different cell lineages for optimal healing of bone defects (Konnecke et al., 2014; Croes et al., 2015, 2016; Kovach et al., 2015; Bastian et al., 2016; Loi et al., 2016b; El Khassawna et al., 2017; Pajarinen et al., 2019).

Given the fact that the first step in bone formation is inflammation, it is logical to concentrate on the cell lineages that have been shown to be most impactful to inflammation in the context of bone healing (Marsell and Einhorn, 2011). As outlined below, pro-inflammatory mediators from cells of the innate immune system including macrophages, mast cells, polymorphonuclear leukocytes and others are critical initially to preconditioning, priming and activating MSCs into an immunomodulatory and pro-reconstructive phenotype (Kouroupis et al., 2019). Furthermore, MSCs possess Toll-like receptors which are important to the specific pro- or anti-inflammatory phenotype of MSCs (Delarosa et al., 2012; Najar et al., 2017; Kouroupis et al., 2019). In this respect, interactions between macrophages and MSCs are the prototype for studying innate immune system-MSC communication. These interactions are highly contextual; although the innate system presents a preprogrammed sequence of events when subjected to an adverse stimulus, the specific interactions occur in the setting of the local physical, chemical and biologic microenvironment characteristic of a specific organ system (Kouroupis et al., 2019). Thus, different cell lineages provide distinctive signals to MSCs, and alter their function according to local cues. For example, within the hematopoietic cell niche, T cell subpopulations interact with MSCs to determine the balance between myeloid differentiation and adipogenesis (Najar et al., 2018). With respect to bone, T regulatory cells (Tregs) have been noted to play a significant role in regulating MSC differentiation and osteoclast function (Li et al., 2018). Other interactions between T and B cell subsets and MSCs modulate the proliferation and differentiation of MSCs, affecting bone formation and remodeling (Konnecke et al., 2014; Ono et al., 2016). These complex interactions have fostered the field of osteoimmunology in which innate and adaptive immune cells interact with cells of the MSC-osteoblast-osteocyte lineage to regulate bone healing and remodeling (Kovach et al., 2015; Ono and Takayanagi, 2017).

## Methods to Enhance the Function of Harvested Cells (Figure 2)

### Preconditioning of MSCs With Biologics

MSCs engage in crosstalk with other cells of different lineages, and in this way, are exposed to biological cues from the local microenvironment and other regional tissues. This intercellular crosstalk is accomplished directly or indirectly through processes that introduce MSCs to signals and byproducts from neighboring cells. This may occur directly via cell-to-cell contact, or indirectly by exposure to secreted cytokines, chemokines, exosomes, or other substances that interact with receptors or through other signaling mechanisms (e.g., by phagocytosis) by MSCs (Zhang et al., 2019).

**Figure 2:**
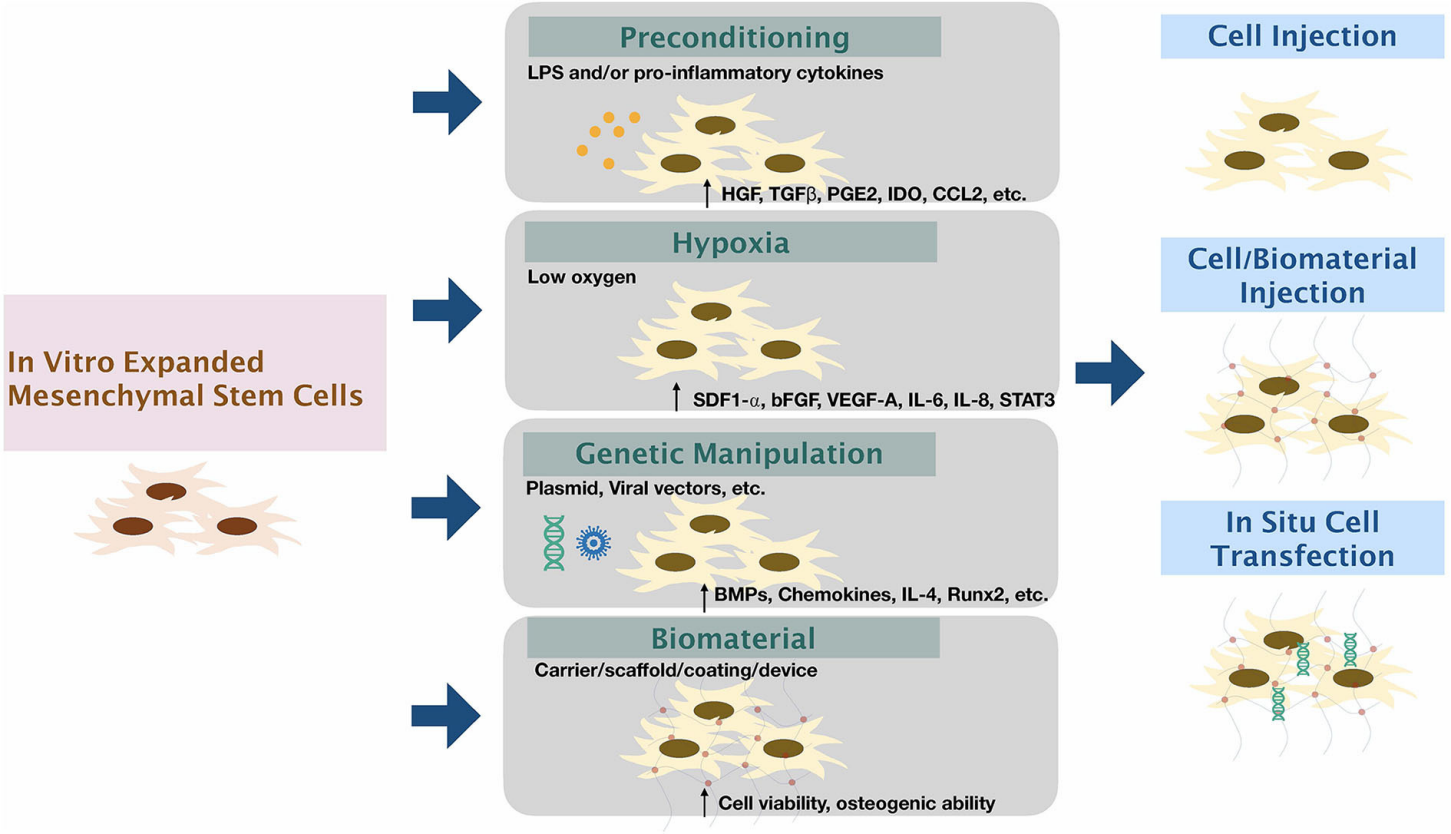
Modifying mesenchymal stem cells to enhance therapeutic functions. The expanded mesenchymal stem cells can be modified via (1) Preconditioning by stimulation by inflammatory factors/substances; (2) Exposure of cells to low oxygen tension; (3) Genetic manipulation using viral or non-viral vectors; and (4) using biomaterial carriers/scaffolds/coatings/devices along or in combination with other methods to enhance the function of MSCs.

Macrophages are much more than phagocytic cells within the innate immune system. Indeed, macrophages are versatile cells with numerous functions and capabilities with respect to immunomodulation and tissue regeneration; this is due to the macrophage's phenotypic plasticity that is responsive to local biological and mechanical signals and cues (Mantovani et al., 2013). MSCs and macrophages have a particularly intricate bidirectional system of interaction. The activation or priming of MSCs by macrophages is one such example. Pro-inflammatory substances such as Tumor Necrosis Factor alpha (TNFα), Interferon gamma (IFNγ), Interleukin-1 (IL-1) and other substances prime MSCs into a state facilitating the resolution of inflammation, vasculogenesis, and tissue healing/reconstruction (Kim and Hematti, 2009; Glass et al., 2011; Carvalho et al., 2013; Croes et al., 2015; Karnes et al., 2015). One paradigm emanates from the concept of polarization of MSCs into MSC1 and MSC2 phenotypes (Waterman et al., 2010). MSC1 cells release primarily pro-inflammatory mediators whereas MSC2 cells are primarily immunosuppressive/pro-reconstructive. Thus, exposure of “uncommitted” MSCs in the stroma to inflammatory mediators (cytokines, chemokines etc.) from macrophages, polymorphonuclear leukocytes (PMNs) and other cells polarizes the MSC into an immunomodulating, tissue regenerative phenotype. Teleologically, this system of checks and balances would tend to preserve the integrity of the local tissues when faced with potentially lethal stimuli, which if persistent, would overwhelm the organism.

Preconditioning of MSCs with IFNγ upregulates many growth factors [such as hepatocyte growth factor (HGF), Transforming Growth Factor beta (TGFβ), and others], Prostaglandin E2 (PGE2), Indoleamine 2,3-dioxygenase (IDO)-an immune checkpoint molecule, the chemokine CCL2 (also known as Macrophage Chemotactic Protein 1 or MCP-1), suppresses CD4^+^ and CD 8^+^ T cell and NK cell proliferation, and polarizes macrophages from a pro-inflammatory M1 to an anti-inflammatory M2 phenotype (de Witte et al., 2015; Lin et al., 2017a; Philipp et al., 2018; Noronha Nc et al., 2019). Preconditioning of MSCs with TNFα leads to similar though less pronounced results compared to IFNγ (de Witte et al., 2015; Noronha Nc et al., 2019). Some of the reported studies have used adipose-derived MSCs (ASCs) whereas others have used bone marrow derived MSCs (BM-MSCs). Results of preconditioning with TNFα on bone regeneration have varied for different cell sources. However, the majority of studies suggests that preconditioning with low dose TNFα enhances immunomodulation and osteogenesis (Lu Z. et al., 2013; Croes et al., 2015; Bastidas-Coral et al., 2016; Lu et al., 2017). However, Lin et al. found that neither IFNγ nor TNFα pre-conditioning, alone or in combination, promoted osteogenesis using murine primary MSCs. In contrast, TNFα in combination with lipopolysaccharide promoted alkaline phosphatase activity and new bone formation *in vitro* (Lin et al., 2017a).

Bastidas-Coral and colleagues explored how exposure to the cytokines TNF-α, IL-6, IL-8, IL-17F, and IL-4 affected the proliferation and osteogenic differentiation of human adipose stem cells (hASCs) *in vitro* for 72 h (Bastidas-Coral et al., 2016). The different cytokines had variable effects on different markers of bone formation including alkaline phosphatase expression and bone nodule formation. Interestingly, addition of IL-6 increased both of the above markers in dramatic fashion and was thought to be a candidate for future studies on bone repair.

It is clear that preconditioning of MSCs with pro-inflammatory factors from macrophages and other cells alters MSC phenotype, and generally supports bone formation. The reverse is true as well: byproducts from MSCs affect macrophage phenotype, generally providing a faciliatory stimulus for bone formation (Prockop et al., 2010; Pajarinen et al., 2019). In fact, MSCs were found to provide an important immunomodulatory function on inflammatory processes via iNOS and a COX2 dependent pathway to enhance PGE2 production (Nemeth et al., 2009; Maggini et al., 2010). These events increase IL-10 secretion by macrophages through binding with Prostaglandin E receptors (EP)2 and EP4. The effects of MSCs on macrophages and other cells is of immense importance; further details can be found in other publications (Kim and Hematti, 2009; Cho et al., 2014; Pajarinen et al., 2019).

Platelet Rich Plasma (PRP) is a derivative of blood, and consists of plasma, plasma proteins, and in addition, the contents of the alpha granules of activated platelets which contain growth factors, cytokines, chemokines and other substances. *In vitro* studies have shown that PRP increases the proliferation and differentiation of MSCs and ASCs, important cells for the formation of bone (Kasten et al., 2006; Vogel et al., 2006; McLaughlin et al., 2016). A recent systematic review on the subject, which summarized the preclinical and clinical data on the subject concluded that the utility of PRP for bone healing is still controversial. Although *in vitro* studies are suggestive of potential utility of PRP in facilitating bone healing, the many different methods and techniques of preparation and use of PRP, including numerous animal models and methods of evaluation have yielded research literature of low quality (Roffi et al., 2017). Clinical studies in humans on the use of PRP for the treatment of acute fractures, delayed unions and non-unions were also guarded (Roffi et al., 2017). The majority of the studies that the authors reviewed led them to the finding that key aspects that could potentially affect the final outcome including platelet concentrations, leukocyte components, activation modality and others were either inconsistent or not stipulated in the publication (Roffi et al., 2017). The authors' final conclusion was the following: “Overall, the available literature presents major limitations in terms of low quality and extreme heterogeneity, which hamper the possibility to optimize PRP treatment and translate positive preclinical findings on its biological potential to favor bone healing into a real clinical benefit.” (Roffi et al., 2017). A recent study examining the effect of PRP on a healing osteotomy for anterior cruciate deficiency in dogs found no significant effect of PRP on healing of the osteotomy as assessed using radiographs, ultrasound, or MRI (Franklin et al., 2017). This skepticism is echoed in other reviews of the use of PRP in fractures and non-unions in humans (Nauth et al., 2015; Marongiu et al., 2020). A systematic review and meta-analysis of the use of PRP in oral surgery concluded that the studies were of low quality; in periodontal defects the addition of PRP may have a slight benefit (Franchini et al., 2019). It is clear that further well-designed prospective studies with appropriate controls need to be performed prior to widespread use of PRP as an adjunct for bone healing.

Numerous cytokines, growth factors, pharmacological agents and chemicals have been shown to activate or prime MSCs for various applications in different organ systems (Noronha Nc et al., 2019). Some of these methods and applications are relevant to bone. The reader is referred to these publications for further information.

### Exposure of Cells to Low Oxygen Environments

Though ambient conditions (i.e., room air) contain ~21% oxygen (O_2_) and *in vitro* cell culture O_2_ tension is about 18%, the physiological O_2_ tension in bone marrow and in the peripheral tissues *in vivo* is only 1–7% (Mohyeldin et al., 2010; Wagegg et al., 2012). This low oxygen tension is also seen locally after musculoskeletal trauma and fracture, and in other instances of inflammation. Hypoxic conditions locally are important determinants of the subsequent function of osteogenic and vasculogenic progenitor cells (Volkmer et al., 2010; Garcia-Sanchez et al., 2019). The cellular response to hypoxia is generally controlled by the transcription factor hypoxia-inducible factor (HIF)-1. HIF-1 is activated under hypoxic conditions and controls numerous cellular processes including the method of cellular metabolism (favoring anaerobic glycolysis over aerobic oxidative metabolism), angiogenesis, and erythropoiesis. There has been some controversy concerning the effects of hypoxia on MSCs and their downstream lineage cells. Currently, with reference to human cells, hypoxia has been shown to stabilize the immunophenotype of MSCs and shifts their differentiation from the adipogenic to the osteogenic lineage in a HIF dependent manner (Wagegg et al., 2012). Furthermore, hypoxia induces the secretion of stroma cell-derived factor 1 alpha (SDF1-α or CXCL12), a potent chemotactic factor for MSCs and other cells; SDF1-α functions together with the chemokine receptor CXCR4 (Park et al., 2017). Numerous other anti-inflammatory and pro-reconstructive cytokines and chemokines are also upregulated by hypoxia (Gabrielyan et al., 2017; Quade et al., 2020). In mice, both MSCs and the media from bone marrow-derived MSCs cultured under hypoxic conditions demonstrated increased amounts of basic fibroblast growth factor (bFGF), vascular endothelial growth factor A (VEGF-A), interleukin 6 (IL-6), and interleukin 8 (IL-8), and enhanced the proliferation and migration of endothelial cells and other cells including macrophages (Chen et al., 2014). Osteogenesis and angiogenesis are also controlled by the transcription factor Signal Transducer and Activator of Transcription 3 (STAT3), an important regulator of bone homeostasis (Yu et al., 2019). Using murine MSCs *in vitro* and a femoral defect model, hypoxia was shown to upregulate the phosphorylation of STAT and was important to the healing of a bone defect. Interestingly, the duration of hypoxia was critical to osteogenesis and angiogenesis, with 3 days of exposure being optimal (Yu et al., 2019). Other studies in animals have confirmed hypoxic preconditioning as a method to enhance osteogenesis, angiogenesis and bone healing, even in aged animals (Fan et al., 2015; Zhang et al., 2018). Hypoxic preconditioning of MSCs has also been suggested as a treatment for osteonecrosis of the femoral head, where local hypoxic conditions are present (Ciapetti et al., 2016).

### Genetic Manipulation of Cells

Gene therapy was originally conceived as a treatment for specific intractable genetically-based diseases in which the gene to be transferred was either missing or significantly altered, resulting in a phenotype that was deficient in a clinically significant manner. This classic approach to gene therapy might be applied to Mendelian based diseases such as hemophilia, sickle cell disease, Gaucher's disease, osteogenesis imperfecta, and others. Current definitions of gene therapy have changed due to recent technical advances in genetic engineering of cells. Presently, potential treatments include vector-delivered gene therapy, gene-modified cell therapy and gene editing (Salzman et al., 2018). Salzman et al. summarized the clinical aim of these treatment concepts succinctly: “Rather than treating disease symptoms, gene therapy can address the root causes of genetic diseases by modifying expression of a patient's genes or by repairing or replacing abnormal genes.” (Salzman et al., 2018).

Although non-union of fractures and deficient healing of bone defects are usually not life-threatening, these conditions often cause significant pain and functional impairment. In this regard, methods to expedite the healing of bone defects and fractures has merit. Thus, the concept of gene therapy has entered the realm of bone tissue engineering and repair (Evans, 2010; Lu C. H. et al., 2013; Balmayor and van Griensven, 2015; Evans and Huard, 2015; Atasoy-Zeybek and Kose, 2018; Ball et al., 2018; Betz et al., 2018; Bougioukli et al., 2018; Shapiro et al., 2018; Freitas et al., 2019). The advantages of gene delivery include the persistent release of a growth factor(s) or other substance(s) over a period of weeks to months, which is generally longer than with local protein delivery devices. Some of the disadvantages of gene delivery include less control over the timing and dose of delivery of a biological agent, safety concerns such as the potential for unintended adverse effects, including carcinogenicity on non-target cells and limitations regarding irreversibility of treatment.

On a practical level, gene therapy has been accomplished using different methods including: non-viral chemical and physical methods to deliver DNA or microparticles into cells, gene activated matrices (GAMs) or scaffolds that enable the slow release of genetic material to the surrounding cells, the use of viral vectors to transfer genes into cells *in vivo*, and genetically engineered autologous or allogeneic cells *ex vivo* with subsequent delivery of these cells *in vivo* (Atasoy-Zeybek and Kose, 2018; Shapiro et al., 2018). All of these methods have been used in preclinical studies to facilitate bone formation, and some are in early clinical trials (see summaries in references D'Mello et al., 2017; Atasoy-Zeybek and Kose, 2018; Betz et al., 2018; Freitas et al., 2019). A variety of genes that have been delivered in various ways including BMP-2, BMP-4, BMP-7, HIF-1, lysosomal integral membrane protein-1 (LIMP-1), PTH 1-34, PDGF-B, VEGF, caALK2 (a BMP receptor), Runx2, RANKL, and combinations thereof (D'Mello et al., 2017; Atasoy-Zeybek and Kose, 2018).

Non-viral based vector therapy is usually accomplished using circular plasmid dsDNA. This method is generally inexpensive, relatively fast, and is accomplished in one step via direct injection, with less concern regarding multistep contamination seen with *ex vivo* methods (Atasoy-Zeybek and Kose, 2018). However, there are concerns regarding the generally low levels of transfection of the specific target cells, unintended transfection of other cells, the short duration of gene expression *in vivo* and therefore the low levels of protein expression. Techniques that have been employed include electroporation, sonoporation, microinjection and other mechanical and biological methods.

Gene activated matrices (GAMs) use a three-dimensional scaffold which is porous and usually biodegradable to transfer plasmid DNA (pDNA) to the local environment (D'Mello et al., 2017; Atasoy-Zeybek and Kose, 2018). The pDNA is transfected into neighboring cells that infiltrate the GAM; these cells subsequently produce the desired protein. The method is relatively inexpensive and easy to produce even on a large scale, is locally effective in producing the desired biological factors, and generally demonstrates low toxicity, pathogenicity, mutagenicity, carcinogenicity and immunogenicity. Commonly used scaffolds include collagen, gelatin, alginate, chitosan, and silk. More mechanically stable structures, such as allograft bone, calcium-based ceramics, and combinations of polymers with/without calcium-based compounds have been used. The physical and chemical properties of the scaffold are key to cellular infiltration and attachment, as well as gene delivery. The GAM technique has been successfully used in pre-clinical studies to release various growth factors (such as VEGF, BMPs, and others) in a balanced manner to optimize the desired effects and limit toxicity. Recently, the use of RNA-based scaffolds for delivery of mRNA, miRNA, and siRNA has been described (Leng et al., 2020). This novel technology can deliver one or more RNA factors simultaneously to the tissues locally. However, the exact cells that are transfected cannot be precisely controlled.

Viral vectors are employed to aid in the transmission of DNA into the host cell (Evans and Huard, 2015; Ball et al., 2018). The virus is altered to make it less virulent and pathogenic, and the genetic sequence of the intended biological factor to be delivered is added to the viral DNA to form a recombinant structure. This recombinant viral vector can be used as a stand-alone circular plasmid (episome) for short term effects or can be integrated into the DNA of the host cell for longer term expression (Balmayor and van Griensven, 2015; Atasoy-Zeybek and Kose, 2018; Ball et al., 2018; Betz et al., 2018). Some of the considerations for viral-associated gene therapy include the capacity of the virus for packaging DNA, the efficiency of transduction and gene expression, the desired timing and duration of gene activity, the target cell for potential gene incorporation, the complexity and cost of the construction, the regulation and monitoring of gene and protein expression, whether the virus affects dividing and non-dividing cells alike, and how toxic/mutagenic/immunogenic will the construct be for the host. The most commonly used viral vectors include adenovirus, adeno-associated virus (AAV), lentivirus, and retrovirus. Each of these has different characteristics, risks and benefits (Evans and Huard, 2015; Atasoy-Zeybek and Kose, 2018). Of these, adenovirus has been the most commonly used for bone healing, although they are highly immunogenic and evoke a host response that often limits protein expression (Evans, 2010; Evans and Huard, 2015; Atasoy-Zeybek and Kose, 2018; Bougioukli et al., 2018).

*Ex vivo* genetically engineered autologous or allogeneic cells for *in vivo* cell delivery have been used for several decades in preclinical studies (Balmayor and van Griensven, 2015; Ball et al., 2018; Betz et al., 2018). Autologous cells are harvested from bone marrow or other sources (e.g., ADSCs, muscle cells etc.), selected out (if desired), expanded and then genetically manipulated with insertion of genes using viral or non-viral methods. Allogeneic cells, though less commonly used, are generally MSCs because of their relatively immune-privileged status to the host (although this is controversial) (Berglund et al., 2017; Kiernan et al., 2018). All of the considerations outlined above concerning viral non-cellular infection are relevant to viral cellular infection as well. The cells are transduced *ex vivo* and then implanted into the bone defect or fracture gap in orthopaedic applications. Adenoviral based constructs are the most commonly used viral agents, but other vectors have their proponents (Balmayor and van Griensven, 2015; Ball et al., 2018; Betz et al., 2018). Growth factors relevant to osteogenesis including BMP-2, BMP-4, BMP-7, Epstein-Barr virus latent membrane protein 1 (LMP-1) and others have been inserted into healing defects with some success. Concerns regarding safety and efficacy, as outlined above for viral vectors are applicable. Furthermore, *ex vivo* transfection is rather complex, laborious, time-consuming and expensive. To control the delivery spatially and temporally, novel concepts have been introduced including aptamers which are oligonucleotide or peptide molecules that bind to specific molecules on targeted cells, on-off switches that modulate gene expression (e.g., TET-on/TET-off systems with control by the administration of doxycycline), sensing receptors linked with effector molecules that control subsequent gene expression in a negative feedback loop and the use of tissue specific promoters (Balmayor and van Griensven, 2015).

Can the process of *ex vivo* viral gene infection be linked to *in vivo* cell administration in a more expeditious manner? Lieberman's group has performed extensive studies in rodents with human cells to examine the over-expression of BMP-2 using ASCs and BM-MSCs to enhance bone healing. Recently, they showed that lentiviral transduction of the BMP-2 gene into human mononuclear bone marrow cells using a “next day” or overnight protocol was less effective than the standard “two-step” tissue expansion approach in healing of a rat critical sized femoral defect (Bougioukli et al., 2019). However, both approaches showed improved new bone formation compared to the controls using plain radiographs, microCT imaging, and histomorphological analysis.

Acute inflammation is the first stage of fracture healing (Gerstenfeld et al., 2003; Loi et al., 2016a). Healing of fractures and bone defects cannot proceed through the typical course of biological events if acute inflammation persists beyond several days to a week. Chronic inflammation and fibrosis are associated with non-union of fractures and decreased healing of bone defects. These facts suggest opportunities for genetic manipulation of the microenvironment of the fracture gap and chronic bone defect. One strategy is to encourage migration of cells into the hematoma, non-union or defect site via the local delivery of genetically altered cells that overexpress key chemokines for MSCs and vascular progenitor cells (Herrmann et al., 2015). Some of the chemokines which have been used for this and related purpose (such as osteoporosis) include Stromal Cell-derived Factor 1 (SDF-1), CCL7 (Monocyte Chemotactic Protein 3 or MCP-3), and others (Lien et al., 2009; Herrmann et al., 2015). Growth factors such as BMP-2 and PDGF and others have chemotactic properties in addition to their direct osteogenic and vasculogenic effects (Lu C. H. et al., 2013; Chen et al., 2015; Bougioukli et al., 2019).

Our laboratory's approach to regulate the healing of bone defects is centered on the modulation of the inflammatory response. Interleukin-4 is an anti-inflammatory cytokine that facilitates the resolution of inflammation and promotes tissue regeneration (Loi et al., 2016a,b). We have generated several genetically engineered constructs that over-express IL-4 to facilitate the healing of chronic bone defects. These include transduced murine bone marrow-derived mesenchymal stromal cells (MSCs) that are NF-κB responsive and IL-4 over-expressing, or contain constitutively active IL-4 expression lentiviral vectors (Lin et al., 2017b, 2018a,b). These constructs have been shown to produce clinically significant amounts of IL-4 either continuously, or only when NF-κB is upregulated in a negative-feedback loop. These constructs change M1 pro-inflammatory macrophages into an M2 anti-inflammatory pro-reconstructive phenotype and have been shown to reverse the suppression of bone formation by an adverse stimulus: contaminated polyethylene particles. The genetically modified cells showed *in vivo* survival similar to the vector only controls, and a significant biological effect (increased bone mineral density) for at least 4 weeks when implanted into the bone marrow cavity. NFκB sensing IL-4 secreting MSCs appear to function as an “on demand” drug delivery system to modulate chronic inflammation. Current efforts are focused on constructs to modulate acute inflammation and cellular chemotaxis.

As outlined above, there appears to be numerous potential opportunities for the use of gene therapies to facilitate bone healing and mitigate chronic inflammation; however, these therapies are not yet FDA approved, and must first demonstrate obligatory safety and efficacy profiles and show cost effectiveness, in order to be used in clinical practice.

### Other Techniques to Enhance the Function of MSCs

Bone healing is dependent on both biological and mechanical cues and the local environment to which it is subjected. When composite grafting techniques are used i.e., a material together with MSCs or cell aspirate, the physical, chemical and material properties of the carrier/scaffold/coating/device used will determine, in part, the phenotype of the cells (Nava et al., 2012; Luangphakdy et al., 2013; Hanson et al., 2014; Chen et al., 2019). Although a detailed discussion of the physical, chemical and material determinants affecting MSC phenotype is beyond the scope of this review, some general points can be made.

In tissue engineering applications for the healing of bone defects, various biomaterials are often used in conjunction with MSCs. These materials are composed of different hydrogels and polymers, mineralized proteins, ceramics, porous metals etc. In clinical scenarios relevant to healing of bone defects, cell fate is determined in part by numerous properties of the materials used including the composition, morphology, viscosity, stiffness, porosity, topography, surface wettability, surface energy, surface charge, molecular attachments, protein absorption and numerous other factors (Wilson et al., 2005; Luangphakdy et al., 2013; Hanson et al., 2014; Bilem et al., 2016; Chen et al., 2019). For example, in *in vitro* studies, the topographical differences between 2D and 3D culture conditions have been shown to alter the crosstalk between MSCs and macrophages, and their immune profile (Valles et al., 2015). In studies of healing of critical sized bone defects in canines, cancellous allograft bone proved to be the optimal scaffold, compared to numerous other polymers (Luangphakdy et al., 2013). This seems intuitive, given the material architectural and biomechanical similarities of allograft cancellous bone to host bone. For the facilitation of bone healing for critical size defects with insufficient autologous bone, the optimal biomaterial template might be decellularized functionalized cancellous allograft bone, in which the surface is coated with molecules to increase cell attachment preferentially for the MSC-osteoblast and the vascular cell lineages. The processing of this bone could be optimized to ensure that it was of sufficient mechanical strength for the indication proposed. Further basic research in this area would provide exciting opportunities for novel preclinical studies and subsequent translation to the clinic.

## Discussion

Although the majority of fractures heal uneventfully, up to 10% result in delayed or non-union (Thomas and Kehoe, 2020). Moreover, bone defects due to traumatic and non-traumatic etiologies will not heal if the defect is large, and the biological and mechanical environments are unfavorable (Giannoudis et al., 2007). Autologous bone graft is the gold standard for treatment of non-unions, bone defects and other causes of localized deficiencies in bone stock. In fact, autologous bone graft is the second most frequent tissue that is transplanted worldwide, second only to blood transfusion (Campana et al., 2014). However, autologous bone graft may be insufficient in quantity and/or quality to fulfill the requirements for bone union or healing of a large bone defect. Bone graft substitutes with osteoconductive and even osteoinductive capabilities are generally insufficient to heal critical size long bone defects, in part because they do not provide viable cells for osteogenesis and paracrine signaling, and do not simulate the bone microenvironment sufficiently (Fillingham and Jacobs, 2016; Lee et al., 2019).

In an effort to decrease the potential morbidity of open harvesting of autologous bone graft, less invasive procedures have been introduced (Dimitriou et al., 2011b). In a systematic review by Dimitriou et al. 19.37% of patients who underwent bone graft harvesting from the iliac crest had a complication, whereas only 6% of patients who underwent harvesting using the long bone intramedullary reamer-irrigator-aspirator (RIA) device sustained an adverse event. A recent study has shown an even lower complication rate using the reamer-aspirator; the complication rate of 1.76% was accompanied by a prolonged post-operative pain rate of 6.45% (Haubruck et al., 2018). In a prospective randomized comparative study (Level 1), the reamer-aspirator was found to have the same union rate and less donor-site pain compared with iliac crest autograft for long bone non-unions (Dawson et al., 2014). The use of harvested autologous iliac crest cells (rather than bulk pieces of bone graft) for enhancement of bone healing has been known for over 3 decades. Connolly et al. did not concentrate the mononuclear cell component after bone marrow harvest at first, but later realized the benefits of centrifugation in providing a more compact osteogenic pellet (Connolly et al., 1989, 1991; Connolly, 1995).

Concentrated bone marrow aspirate has been used for the treatment of delayed and non-union of fractures, defects due to chronic inflammation, infection, tumor and other bone deficiencies, osteonecrosis and in other applications for healing of bone (Hernigou et al., 2005, 2009). Despite these successes, there remains significant opportunities for enrichment of the concentrated bone marrow aspirate, and in particular, the isolation and augmentation of osteoprogenitor cells and vascular progenitor cells. The purpose of this review is to summarize the current literature regarding biological treatments of MSCs for augmentation of bone healing. It is recognized that other methods, including mechanical, chemical, pharmacological etc., are also putative solutions to this problem; however, restriction to biological treatments alone reveals a substantial amount of literature for focused review and commentary. Three main treatments of MSCs are highlighted: preconditioning also known as priming or activation of MSCs by biological factors, exposure of MSCs to a hypoxic environment, and genetic manipulation of cells.

Preconditioning of MSCs by cytokines, chemokines, and other substances is “natural,” in the sense that this process currently is the paradigm by which the innate immune system and MSC lineage cells interact to resolve the inflammatory response and reconstruct host tissue. Indeed, these events are common to all organ systems, in which continuous cellular crosstalk among inflammatory cells, MSCs and numerous other cell types is the norm. Without this intercellular “on-line” signaling, adverse stimuli would potentially destroy critical tissues, thereby jeopardizing the long-term viability of the organism.

Biologic preconditioning of cells *ex vivo* is evidence based; however, even though the cells may be autologous, the processes involved in preconditioning are more than “minimal manipulation” (U. S. Food and Drug Administration, 2017). This is especially true if the cells are selected and expanded in culture, such as with MSCs. Ongoing *in vitro, in vivo* and restricted clinical trials will hopefully see this technique translate to the clinic. The first step might be isolation and concentration of harvested autogenous iliac crest cells, which are then exposed to a “benign” treatment, perhaps one (or two) pro-inflammatory cytokine(s), prior to washing and local implantation. This would potentially activate both the progenitor cells and innate immune cells alike, the combination of which is more effective than progenitor cells alone (Kovach et al., 2015; Loi et al., 2016b). This first stage preconditioning concept using autologous concentrated but non-expanded cells might also be extended to the use of low oxygen environments, another effective stimulus.

Genetic manipulation of cells and tissues *ex vivo* and *in vivo* has already been instituted clinically on a limited basis, for severe incurable illnesses in which genetically-based diseases are associated with a devastating phenotype. Once the principles of gene therapy are better understood, and the questions related to safety and efficacy are answered more clearly, other less serious but debilitating conditions might be considered. It is in this realm that accelerated bone healing using genetic manipulation of the cells may find a suitable application. Many questions still need to be answered regarding the optimal platform, appropriate dosing and timing, potential immunogenicity, mutagenicity, carcinogenicity, unintended adverse effects on neighboring cells, as well as cost effectiveness of treatment.

## Future Directions

There are major efficacy and regulatory concerns as well as issues related to cost that need to be addressed prior to the widespread introduction and use of new technologies to improve bone healing using modified MSCs and their byproducts. Indeed, some of the issues relevant to even the use of *unmodified* MSCs and their byproducts alone have only recently been posed and reviewed (Diederichs et al., 2013; Marolt Presen et al., 2019; Robb et al., 2019). Exhaustive *in vitro* and *in vivo* preclinical studies in small and large animals must be performed, as what may work in the culture plate and small animals may be quite different from what works in larger animals and humans. There are major issues related to which cell types or lineages are most appropriate for selection, the methods by which these cells will be confirmed, harvested, isolated, expanded, and their purity, potency, stability, and sterility assured. Storage mechanisms for easy access to the end-user and user-friendly delivery mechanisms and technologies must be invented. Importantly, the cells must be shown to be efficacious and safe and cost effective. This should be accomplished by well-designed prospective, randomized studies, with meticulous documentation and oversight, for clear indications in specific populations. Databases for long-term follow-up of biological therapies should be established. Performing novel cutting edge therapies with substantial potential risk for serious life threating diseases such as cancer and end-stage heart disease is one end of the spectrum. Non-union of fractures and bone defects are not life threatening, although their impact on quality of life is often substantial. Nonetheless, eventually graduated clinical trials must be performed according to the principles outlined by groups such as with the IDEAL recommendations and others (McCulloch et al., 2009; Ergina et al., 2013). All of these concerns must be addressed in a very complex regulatory environment, which in the USA has rather strict regulations concerning the modification of cells, which can only be “minimally manipulated” (U. S. Food and Drug Administration, 2017). Finally, cost-effective analyses and value-based health care decision making will be important determinants as to whether these new technologies translate to the clinic, or remain only a subject of scientific inquiry (Burnham et al., 2017).

## Conclusion

Complete healing of fractures and bone defects in specific patient populations and difficult clinical scenarios is still an unmet medical need. Novel approaches and techniques are challenging the belief that autologous bone graft procedures are always the best surgical solutions for obtaining bone union. The harvesting, concentration, and possible manipulation of the phenotype of osteoprogenitor and vascular progenitor cells using preconditioning protocols, exposure to low oxygen environments and genetic manipulation may provide new opportunities for obtaining healing of bone, while minimizing the morbidity associated with open bone grafting. However, these new technologies have substantial scientific, regulatory, and financial hurdles that must be overcome prior to widespread use.

## Author Contributions

All authors listed have made a substantial, direct and intellectual contribution to the work, and approved it for publication.

## Conflict of Interest

The authors declare that the research was conducted in the absence of any commercial or financial relationships that could be construed as a potential conflict of interest.

## Abbreviations

AAV: adeno-associated virus
ABG: autologous bone graft
ALK: alkaline phosphatase
ASC: Adipose-derived stem cell
bFGF: basic fibroblast growth factor
BM-MSC: bone marrow derived MSC
BMP: bone morphogenetic protein
CCL2: C-C motif ligand 2
CD: cluster differentiation
CFU-F: colony forming unit-fibroblast
COX: cyclo-oxygenase
CXCR: C-X-C motif receptor
DNA: deoxyribonucleic acid
dsDNA: double stranded DNA
EP: prostaglandin E receptor
EPC: endothelial progenitor cells
ECFC: endothelial colony-forming unit
GAMs: gene activated matrices
GMP: Good Manufacturing Practices
hASC: human adipose stem cells
HGF: hepatocyte growth factor
HIP: hypoxia-inducible factor
hSSC: human skeletal stem cell
IDO, indoleamine 2: 3-dioxygenase
IL: interleukin
iNOS: inducible nitric oxide synthase
ISCT: International Society for Cell and Gene Therapy
IFNγ: interferon gamma
LIMP-1: lysosomal integral membrane protein-1
LMP-1: latent membrane protein 1
MCP-1: macrophage chemotactic protein 1
MSC, mesenchymal stromal cell. In some of the text: specific quoted authors have referred to MSC as mesenchymal stem cell. Please refer to this usage in the text below
NF-κB: nuclear factor-κB
NK cells: natural killer cells
pDNA: plasmid DNA
PDGF: platelet-derived growth factor
PG: prostaglandin
PRP: platelet rich plasma
PTH: parathyroid hormone
RANKL: receptor activator of nuclear factor-κB ligand
RBC: red blood cell
RNA: ribonucleic acid
Runx2: runt-related transcription factor 2
SDF: stroma cell-derived factor
STAT3: Signal Transducer and Activator of Transcription 3
SVF: stromal vascular fraction
TET: tetracycline
TGFβ: transforming growth factor beta
TNFα: tumor necrosis factor alpha
Tregs: T regulatory cells
VEGF: vascular endothelial growth factor.
